# Evaluation of plasma vitamin E and development of proteinuria in hypertensive patients

**DOI:** 10.2478/jtim-2023-0004

**Published:** 2023-04-01

**Authors:** Panpan He, Huan Li, Yuanyuan Zhang, Yun Song, Chengzhang Liu, Lishun Liu, Binyan Wang, Huiyuan Guo, Xiaobin Wang, Yong Huo, Hao Zhang, Xiping Xu, Jing Nie, Xianhui Qin

**Affiliations:** National Clinical Research Center for Kidney Disease, State Key Laboratory for Organ Failure Research, Division of Nephrology, Nanfang Hospital, Southern Medical University, Guangzhou 510515, Guangdong Province, China; Beijing Advanced Innovation Center for Food Nutrition and Human Health, College of Food Science and Nutritional Engineering, China Agricultural University, Beijing 100083, China; Institute of Biomedicine, Anhui Medical University, Hefei 230032, Anhui Province, China; Shenzhen Evergreen Medical Institute, Shenzhen 518057, Guangdong Province, China; Department of Population, Family and Reproductive Health, Johns Hopkins University Bloomberg School of Public Health, Baltimore 21205, MD, USA; Department of Cardiology, Peking University First Hospital, Beijing 100034, China; AUSA Research Institute, Shenzhen AUSA Pharmed Co Ltd, Shenzhen 518057, Guangdong Province, China

**Keywords:** plasma vitamin E, α-tocopherol, proteinuria, patients with hypertension

## Abstract

**Background:**

The prospective relationship between plasma vitamin E levels and proteinuria remains uncertain. We aimed to evaluate the association between baseline plasma vitamin E levels and the development of proteinuria and examine any possible effect modifiers in patients with hypertension.

**Methods:**

This was a post hoc analysis of the renal sub-study of the China Stroke Primary Prevention Trial (CSPPT). In total, 780 participants with vitamin E measurements and without proteinuria at baseline were included in the current study. The study outcome was the development of proteinuria, defined as a urine dipstick reading of a trace or ≥ 1+ at the exit visit.

**Results:**

During a median follow-up duration of 4.4 years, the development of proteinuria occurred in 93 (11.9%) participants. Overall, there was an inverse relationship between plasma vitamin E and the development of proteinuria (per standard deviation [SD] increment; odds ratio [OR]: 0.73, 95% confidence interval [CI]: 0.55–0.96). Consistently, when plasma vitamin E was assessed as quartiles, lower risk of proteinuria development was found in participants in quartiles 2–4 (≥ 7.3 μg/mL; OR: 0.57, 95% CI: 0.34–0.96) compared to those in quartile 1. None of the variables, including sex, age, and body mass index, significantly modified the association between vitamin E and proteinuria development.

**Conclusion:**

There was a significant inverse association between plasma vitamin E levels and the development of proteinuria in patients with hypertension. The results were consistent among participants with different baseline characteristics.

## Introduction

Proteinuria is a consequence of increased albumin leakage due to increased intraglomerular pressure and glomerular capillary wall permeability.^[1,2]^ In a cross-sectional survey of a nationally representative sample of Chinese adults, the overall prevalence of proteinuria was 9.4%,^[3]^ underscoring the substantial population burden of proteinuria. As a biomarker of microvascular and macrovascular endothelial dysfunction,^[2]^ proteinuria is not only a marker for renal disease,^[4]^ but also a strong predictor of the risk of cardiovascular events and mortality.^[5,6]^ Moreover, an individual patient meta-analysis reported that a reduction in proteinuria was associated with a lower risk of end-stage renal disease or death.^[7]^ However, traditional risk factors, such as primary kidney disease, hypertension, diabetes, and obesity, do not account for all risks of proteinuria.^[4]^ Therefore, there is an urgent need to identify important and modifiable risk factors for proteinuria to prevent its development and related diseases. To this end, the association between vitamins and the risk of proteinuria in diabetes or kidney disease patients has received considerable attention.^[8,9]^

Vitamin E is an essential, fat-soluble vitamin. It is abundantly present in seeds and edible oils.^[10]^ Once ingested, vitamin E is taken up by intestinal cells and released into circulation in chylomicrons.^[11]^ Vitamin E is a potent antioxidant with strong anti-inflammatory properties.^[11]^ As such, it is speculated that a link may exist between vitamin E and proteinuria. Indeed, in streptozotocin-induced diabetic rats,^[12]^ antioxidant treatment with vitamin E normalized renal dysfunction, such as albuminuria and glomerular hypertension. However, data on the association between vitamin E and proteinuria are inconsistent between cross-sectional studies^[13,14,15]^ or clinical trials,^[16,17,18,19,20,21,22,23]^ and the prospective association between vitamin E and the development of proteinuria remains uncertain.

Therefore, to address the aforementioned gap, our current study, a post hoc analysis of the renal sub-study of the China Stroke Primary Prevention Trial (CSPPT), aimed to evaluate the prospective association between plasma vitamin E levels and the development of proteinuria and examine any possible effect modifiers among patients with hypertension.

## Materials and methods

### Study participants

The study design, methods, and major results of the CSPPT^[24,25]^ and the renal sub-study of the CSPPT^[26,27]^ have been previously described in detail. Briefly, the CSPPT was a multi-community, randomized, double-blind, controlled trial conducted between May 19, 2008 and August 24, 2013 in 32 communities in China. Eligible participants were men and women aged 45–75 years with hypertension, defined as seated, resting systolic blood pressure (SBP) ≥ 140 mmHg or diastolic blood pressure (DBP) ≥ 90 mmHg at both the screening and recruitment visits or those who were taking antihypertensive medication. The major exclusion criteria were a history of physician-diagnosed stroke, myocardial infarction (MI), heart failure, post-coronary revascularization, and/or congenital heart disease. The CSPPT found that among adults with hypertension in China without a history of stroke or MI, the combined use of enalapril and folic acid, compared to enalapril alone, significantly reduced the risk of first stroke.^[24]^

The renal sub-study of the CSPPT (*n* = 15,104) enrolled eligible CSPPT participants from 20 communities in Jiangsu province, excluding those with an estimated glomerular filtration rate (eGFR) < 30 mL/min/1.73m^2^ or with missing eGFR at baseline. The CSPPT renal sub-study reported that enalapril-folic acid therapy, compared to enalapril alone, can significantly delay the progression of chronic kidney disease (CKD) in patients with mild-to-moderate CKD.^[26]^

A total of 1500 baseline participants from the CSPPT were randomly selected for the vitamin E measurements. The current study included participants from the renal sub-study of the CSPPT who had vitamin E measurements and had complete data on urine protein status at both the baseline and exit visits, as well as urine dipstick readings of none at baseline (Supplemental Figure 1).

Both the parent study and this study were approved by the ethics committee of the Institute of Biomedicine, Anhui Medical University, Hefei, China (FWA assurance number: FWA00001263). All participants provided written informed consent.

### Intervention and follow-up

Eligible participants were randomly assigned, in a 1:1 ratio, to one of two treatment groups: a daily oral dose of one tablet containing 10 mg enalapril and 0.8 mg folic acid (the enalapril-folic acid group) or a daily oral dose of one tablet containing 10 mg enalapril only (the enalapril-only group).

During the trial period, concomitant use of other antihypertensive drugs was allowed if the blood pressure was not properly controlled. The participants were followed up every 3 months. At each visit, blood pressure was measured, the number of pills taken between visits was counted, and concomitant medications and adverse events were recorded.

### Laboratory assays

Serum and spot urine samples were collected from the participants at both the baseline and exit visits. Serum folate and vitamin B12 levels were measured in a commercial laboratory using chemiluminescent immunoassay (New Industrial). Total homocysteine (tHcy), lipid, and glucose levels were measured using automatic clinical analyzers (Beckman Coulter) at the core laboratory of the National Clinical Research Center for Kidney Disease, Nanfang Hospital, Guangzhou, China. Plasma vitamin E (α-tocopherol) was measured using liquid chromatography with tandem quadrupole mass spectrometry (LC–MS/ MS) in a commercial laboratory (Beijing DIAN Medical Laboratory, China). Proteinuria was determined using a dipstick test (Dirui-H100; Jilin, China). eGFR was calculated using the Chronic Kidney Disease Epidemiology Collaboration (CKD-EPI) equation.

### Outcomes

The study outcome was the development of proteinuria, defined as a urine dipstick reading of a trace or ≥ 1+ at the exit visit.

### Statistical analysis

Baseline characteristics are presented as the mean (standard deviation [SD]) for continuous variables or proportions for categorical variables by vitamin E quartiles. Differences in characteristics were compared using analysis of variance (ANOVA), chi-square test, or Fisher’s exact test.

Multivariable logistic regression models were used to examine the relationship between baseline plasma vitamin E levels and the development of proteinuria, with and without adjustments for sex, age, treatment group, body mass index (BMI), SBP, eGFR, total cholesterol (TC), triglycerides (TG), high-density lipoprotein cholesterol (HDL-C), fasting glucose, tHcy, smoking and alcohol drinking status, use of antihypertensive drugs at baseline, and time-averaged SBP during the treatment period.

In the stratified analysis, possible modifications of the association between baseline plasma vitamin E and the development of proteinuria were assessed for the following variables: sex, age (< 60 and ≥ 60 years), BMI (< 24, 24–< 28, and ≥ 28 kg/m^2^), treatment group (enalapril and enalapril-folic acid), SBP (< 160 and ≥ 160 mmHg), tHcy (< 12.4 [median] and ≥ 12.4 μmol/L), TC (< 5.2 and ≥ 5.2 mmol/L), eGFR (< 90 and ≥ 90 mL/min/1.73 m^2^), and fasting glucose (< 5.6, 5.6–< 7.0 mmol/L, and diabetes) at baseline and time-averaged SBP during the treatment period (< 140 and ≥ 140 mmHg). Diabetes was defined as fasting glucose ≥ 7.0 mmol/L, the use of glucose-lowering drugs, or a self-reported history of diabetes.

A two-tailed *P* < 0.05 was considered to be statistically significant in all analyses. The R software, version 3.5.0 (*R* Foundation for Statistical Computing, Vienna, Austria, http://www.R-project.org/) was used for all statistical analyses.

## Results

### Study participants and baseline characteristics

As illustrated in the flowchart (Supplementary Figure 1), 780 participants were included in the final analysis. Participants excluded from the analysis did not differ substantially in their baseline characteristics from those included in the analysis (Supplementary Table 1).

The baseline characteristics of the participants are presented as plasma vitamin E quartiles (Table 1). Participants with higher plasma vitamin E levels tended to have higher TG, TC, HDL-C, fasting glucose, and folate levels and lower frequency of use of antihypertensive and antiplatelet drugs. Moreover, the key baseline characteristics were similar between the two treatment groups (Supplementary Table 2).

**Table 1:** Characteristics of study participants by vitamin E quartiles

Characteristics	Q1 (< 7.3 μg/mL)	Q2 (7.3–< 8.8 μg/mL)	Q3 (8.8–< 11.3 μg/mL)	Q4 (≥ 11.3 μg/mL)	*P*-value
Male, *n* (%)	81 (41.5)	69 (35.4)	61 (31.3)	70 (35.9)	0.211
Age, years	60.0 (7.8)	59.7 (7.7)	58.8 (7.2)	59.6 (7.5)	0.487
BMI, kg/m^2^	25.5 (3.7)	25.2 (3.5)	25.6 (3.6)	25.9 (3.6)	0.271
BP, mmHg					
Baseline SBP	164.9 (19.5)	167.0 (19.3)	165.1 (17.7)	165.9 (20.5)	0.702
Baseline DBP	93.5 (11.7)	93.9 (12.3)	95.0 (11.7)	93.4 (11.5)	0.535
Time-averaged SBP	139.3 (9.9)	138.9 (9.8)	139.4 (10.1)	138.2 (10.6)	0.628
Time-averaged DBP	82.8 (7.1)	83.4 (7.0)	83.5 (7.2)	82.7 (7.1)	0.583
Enalapril-folic acid, *n* (%)	104 (53.3)	91 (46.7)	97 (49.7)	97 (49.7)	0.628
Current smoking, *n* (%)	49 (25.1)	41 (21.0)	33 (16.9)	47 (24.1)	0.257
Current alcohol drinking, *n* (%)	40 (20.5)	38 (19.5)	38 (19.6)	52 (26.7)	0.413
Laboratory results					
Triglycerides, mmol/L	1.5 (0.7)	1.6 (0.8)	1.7 (0.8)	2.0 (1.2)	<0.001
Total cholesterol, mmol/L	5.0 (1.0)	5.5 (1.0)	5.9 (1.0)	6.1 (1.1)	<0.001
HDL-C, mmol/L	1.2 (0.3)	1.3 (0.3)	1.4 (0.4)	1.4 (0.4)	<0.001
Glucose, mmol/L	5.6 (1.0)	5.9 (1.5)	6.0 (1.7)	6.2 (2.4)	0.006
Total homocysteine, μmol/L	14.6 (7.8)	14.6 (8.6)	14.4 (9.0)	14.1 (9.2)	0.946
Folate, ng/mL	7.0 (3.0)	7.6 (2.7)	7.6 (2.9)	8.3 (3.5)	<0.001
Vitamin B12, μg/mL	395.1 (174.9)	390.6 (113.8)	404.8 (150.3)	417.2 (154.2)	0.312
eGFR, mL/min/1.73m^2^	94.0 (10.7)	93.4 (12.4)	95.6 (11.6)	95.8 (12.7)	0.114
Medication use, *n* (%)					
Antihypertensive drugs	106 (54.4)	81 (41.5)	102 (52.3)	88 (45.1)	0.037
Lipid-lowering drugs	1 (0.5)	1 (0.5)	0	0	1.000
Glucose-lowering drugs	2 (1.0)	1 (0.5)	4 (2.1)	4 (2.1)	0.536
Antiplatelet drugs	15 (7.7)	6 (3.1)	11 (5.6)	2 (1.0)	0.008

Variables are presented as mean (SD) or n (%). The sample size in each group is 195. BP: blood pressure; SBP: systolic blood pressure; DBP: diastolic blood pressure; eGFR: estimated glomerular filtration rate; HDL-C: high-density lipoprotein cholesterol; BMI: body mass index.

### Association between plasma vitamin E and the development of proteinuria

During a median of 4.4 years of follow-up, the development of proteinuria occurred in 92 (11.8%) participants. Participants with urine dipstick readings of trace or ≥ 1 + tended to have lower levels of plasma vitamin E compared to those without proteinuria (8.8 ± 2.7 *vs*. 9.6 ± 3.5 μg/mL, *P* = 0.033), and the plasma vitamin E levels were similar for participants with urine dipstick readings of trace (8.7 ± 2.8 μg/mL) or ≥ 1+ (8.9 ± 2.6 μg/mL) (*P* = 0.757) (Supplemental Table 3).

**Figure 1:**
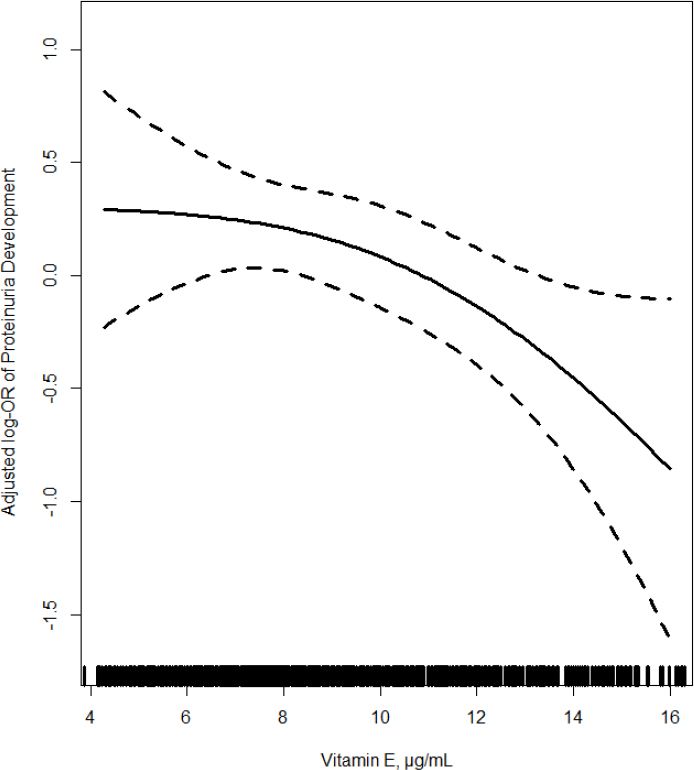
The relationship of plasma vitamin E with the development of proteinuria in patients with hypertension. Adjusted for age, sex, body mass index, treatment group, smoking status, alcohol intake, SBP, eGFR, fasting glucose, total cholesterol, triglycerides, high-density lipoprotein cholesterol, total homocysteine, use of antihypertensive drugs at baseline, and time-averaged SBP during treatment. SBP: systolic blood pressure; eGFR: estimated glomerular filtration rate.

The association between vitamin E levels and the development of proteinuria is shown in Figure 1. Overall, there was an inverse relationship between plasma vitamin E and the development of proteinuria (per SD increment; odds ratio [OR]: 0.73, 95% confidence interval [CI]: 0.55–0.96). Consistently, when plasma vitamin E was assessed as quartiles, a lower risk of proteinuria development (OR: 0.57, 95% CI: 0.34–0.96) was found in participants in quartile 2–4 (≥ 7.3 μg/mL) compared to those in quartile 1 (< 7.3 μg/mL) (Table 2).

**Table 2:** The association between plasma vitamin E and the development of proteinuria

Vitamin E, μg/mL	*n*	Events, *n* (%)	Crude models		Adjusted models*	
				
			OR (95% CI)	*P*-value	OR (95% CI)	*P*-value
Continuous (per SD increment)	780	92 (11.8)	0.77 (0.60, 0.98)	0.033	0.73 (0.55, 0.96)	0.023
Quartiles						
Q1 (< 7.3)	195	31 (15.9)	ref.	—	ref.	—
Q2 (7.3– < 8.8)	195	21 (10.8)	0.64 (0.35, 1.16)	0.138	0.58 (0.32, 1.08)	0.087
Q3 (8.8– < 11.3)	195	20 (10.3)	0.60 (0.33, 1.10)	0.101	0.58 (0.31, 1.11)	0.099
Q4 (≥ 11.3)	195	20 (10.3)	0.60 (0.33, 1.10)	0.101	0.55 (0.28, 1.08)	0.083
Categories						
Q1 (< 7.3)	195	31 (15.9)	ref.	—	ref.	—
Q2–Q4 (≥ 7.3)	585	61 (10.4)	0.62 (0.39, 0.98)	0.042	0.57 (0.34, 0.96)	0.034

*Adjusted for age, sex, body mass index, treatment group, smoking status, alcohol intake, SBP, eGFR, fasting glucose, total cholesterol, triglycerides, highdensity lipoprotein cholesterol, total homocysteine, use of antihypertensive drugs at baseline, and time-averaged SBP during treatment. CI: confidence interval; eGFR: estimated glomerular filtration rate; OR: odds ratio; SBP: systolic blood pressure; SD: standard deviation.

Additionally, during the treatment period, participants with higher plasma vitamin E levels had a lower frequency of diuretic use (Supplementary Table 4). However, further adjustments for diuretic use did not substantially change the results (Supplementary Table 5).

### Stratified analyses by potential effect modifiers

**Figure 2:**
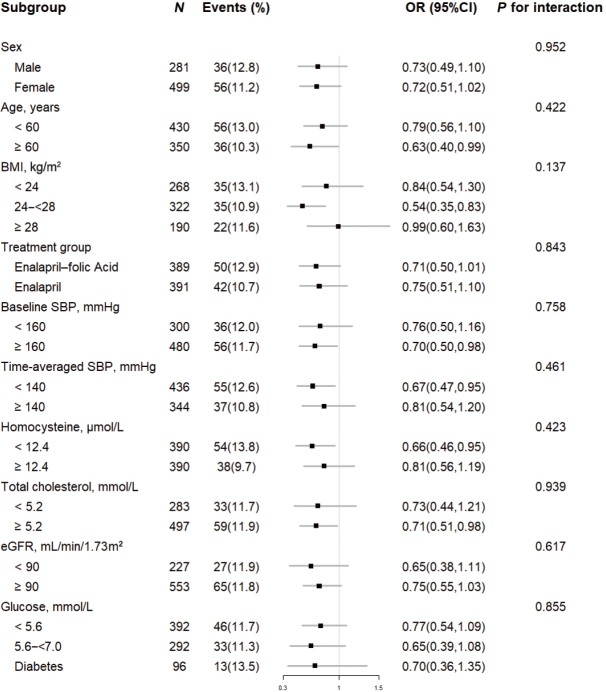
Stratified analyses by potential effect modifiers for the development of proteinuria. Adjusted for age, sex, body mass index, treatment group, smoking status, alcohol intake, SBP, eGFR, fasting glucose, total cholesterol, triglycerides, high-density lipoprotein cholesterol, total homocysteine, use of antihypertensive drugs at baseline, and time-averaged SBP during treatment. Boxes denote ORs and lines represent 95% CIs. SBP: systolic blood pressure; eGFR: estimated glomerular filtration rate; ORs: odds ratios; CIs: confidence intervals.

Stratified analyses were performed to assess the relationship between vitamin E (per SD increment) and proteinuria development in various subgroups (Figure 2). None of the variables, including sex, age, BMI, treatment group, SBP, tHcy, TC, eGFR, and glucose levels at baseline or time-averaged SBP during the treatment period, significantly modified the association between vitamin E and proteinuria development (*P*-value for all interactions > 0.05) (Figure 2).

## Discussion

Our current study demonstrated that plasma vitamin E levels were inversely associated with the development of proteinuria among patients with hypertension. The results were consistent among participants with different baseline characteristics.

Clinical trials have been conducted to evaluate the effect of vitamin E supplementation on proteinuria. However, most of these trials were conducted in patients with diabetes^[16,17,18,19,20]^ and/or renal diseases^[21,22,23]^ and they reported inconsistent effects of vitamin E supplementation on proteinuria. Notably, these trials mainly examined the effects of relatively high vitamin E supplementation in high-risk populations rather than the effects of vitamin E levels in the general population. In addition, studies on the relationship of vitamin E with proteinuria in the general population are inconsistent.^[13,15]^ The International Population Study on Macronutrients and Blood Pressure (INTERMAP) reported that dietary vitamin E intake was inversely related to microalbuminuria in men but not in women.^[15]^ However, Ford *et al*.^[13]^ reported that vitamin E concentrations were not significantly associated with microalbuminuria among adults in the USA. However, these previous studies were cross sectional in design and were not useful in determining the temporal and causal relationships between vitamin E and proteinuria. Therefore, the prospective association between vitamin E levels and proteinuria remains unknown. Our current prospective study provides an opportunity to assess the temporal and dose-response relationship between plasma vitamin E levels and the development of proteinuria. This study had not only baseline vitamin E concentrations, but also all pertinent clinical information and laboratory measurements, including lipids, glucose, and eGFR. Most importantly, this study used urine dipstick tests at both the baseline and exit visits, allowing us to define the development of proteinuria. To the best of our knowledge, this is the first study of this type in a Chinese hypertensive population.

Our findings make new contributions to this field. This prospective study allowed us to assess temporal and dose-response relationships. We found that higher vitamin E levels were significantly associated with a lower risk of proteinuria. Although the biological mechanisms underlying the observed protective relationship between vitamin E and the development of proteinuria remain to be determined, our findings are biologically plausible. Oxidative stress induces cellular apoptosis, glomerular distortion, and regression of podocyte foot processes, with consequent loss of integrity of the glomerular barrier. Reactive oxygen species (ROS) overproduction leads to the increased formation of advanced glycation end products (AGEs), impairs endothelial-derived nitric oxide synthase (e-NOS) activity, and activates polyol, protein kinase C (PKC), and nuclear factor κB (NF-κB) pathways.^[28,29]^ As a small-molecule antioxidant, vitamin E can bind to various active oxidant species (*e.g*., superoxide free radicals) and defend against damage caused by ROS.^[30]^ Animal models have shown that vitamin E supplementation could activate diacylglycerol kinase, an enzyme that, by reducing the circulating levels of diacylglycerol, prevents abnormal activation of PKC and the regression of podocytes.^[31]^ Moreover, researchers have indicated that the administration of vitamin E can enhance the activity of the NO/iNOS system *in vivo*.^[32]^ A previous study also showed that tocotrienol supplementation could inhibit the NF-κB pathway and reduce the inflammatory response.^[33]^ In addition, vitamin E supplementation in type 1 diabetes has been associated with a reduction in the plasma levels of monocyte chemoattractant protein-1 (MCP-1), a chemokine involved in the recruitment of inflammatory cells in the peripheral tissues, which confirms the possible positive effect of vitamin E on inflammation.^[34]^ All of these mechanisms may explain the potential beneficial effects of vitamin E on proteinuria.

The limitations of the present study should be noted. First, we only measured α-tocopherol in plasma and could, therefore, not specify the levels of other different chemical forms of vitamin E (*e.g*., γ-tocopherol and tocotrienol). Second, vitamin E treatment is often combined with vitamin C supplementation to improve hypertensive symptoms in patients. However, we did not have data on vitamin C concentration or supplementation. As such, the association between vitamin C levels and proteinuria in our population remains uncertain. Third, participants’ vitamin E levels were only assessed at baseline, while proteinuria was assessed at baseline and at the exit visit. Fourth, proteinuria was measured using the dipstick test. However, there was a graded association (*P*-trend < 0.001) between dipstick proteinuria and urinary albumin-to-creatinine (ACR) ratio among participants with available urinary ACR values (*n* = 3225) in the CSPPT.^[35]^ White *et al*.^[36]^ found that dipstick testing for proteinuria is both sensitive and specific for macroalbuminuria (ACR ≥ 300 mg/g). The dipstick test for proteinuria is a simple, easy-to-use, widely available, and inexpensive laboratory test. Fifth, this study was conducted on adults with hypertension. The generalizability of these results to populations without hypertension remains to be determined. However, adjustments for blood pressure measurements at baseline and during the trial period did not substantially change the findings. More importantly, our study is a post hoc analysis of the CSPPT. Despite adjusting for a broad set of covariates in the regression models, residual confounding may still exist. As such, our study is only hypothesis generating. Further research on the measurement of other chemical forms of vitamin E and frequent measurements of vitamin E and proteinuria are needed.

In summary, our data suggest an inverse association between plasma vitamin E levels and the development of proteinuria in patients with hypertension. If further confirmed, maintaining optimal vitamin E concentrations may be considered an adjuvant nutritional strategy for the prevention and treatment of proteinuria in hypertensive populations.

## Supplementary Material

Supplementary Material
