# Supplementary material for: Evaluation of plasma vitamin E and development of proteinuria in hypertensive patients
Source: J Transl Int Med. 2023 Apr 1;12(1):78–85. doi: 10.2478/jtim-2023-0004 (PMC10956724; doi:10.2478/jtim-2023-0004)
Supplement: Supplementary file 1 — Supplementary Material [file jtim-2023-0004_sm.pdf]

# Supplementary Material

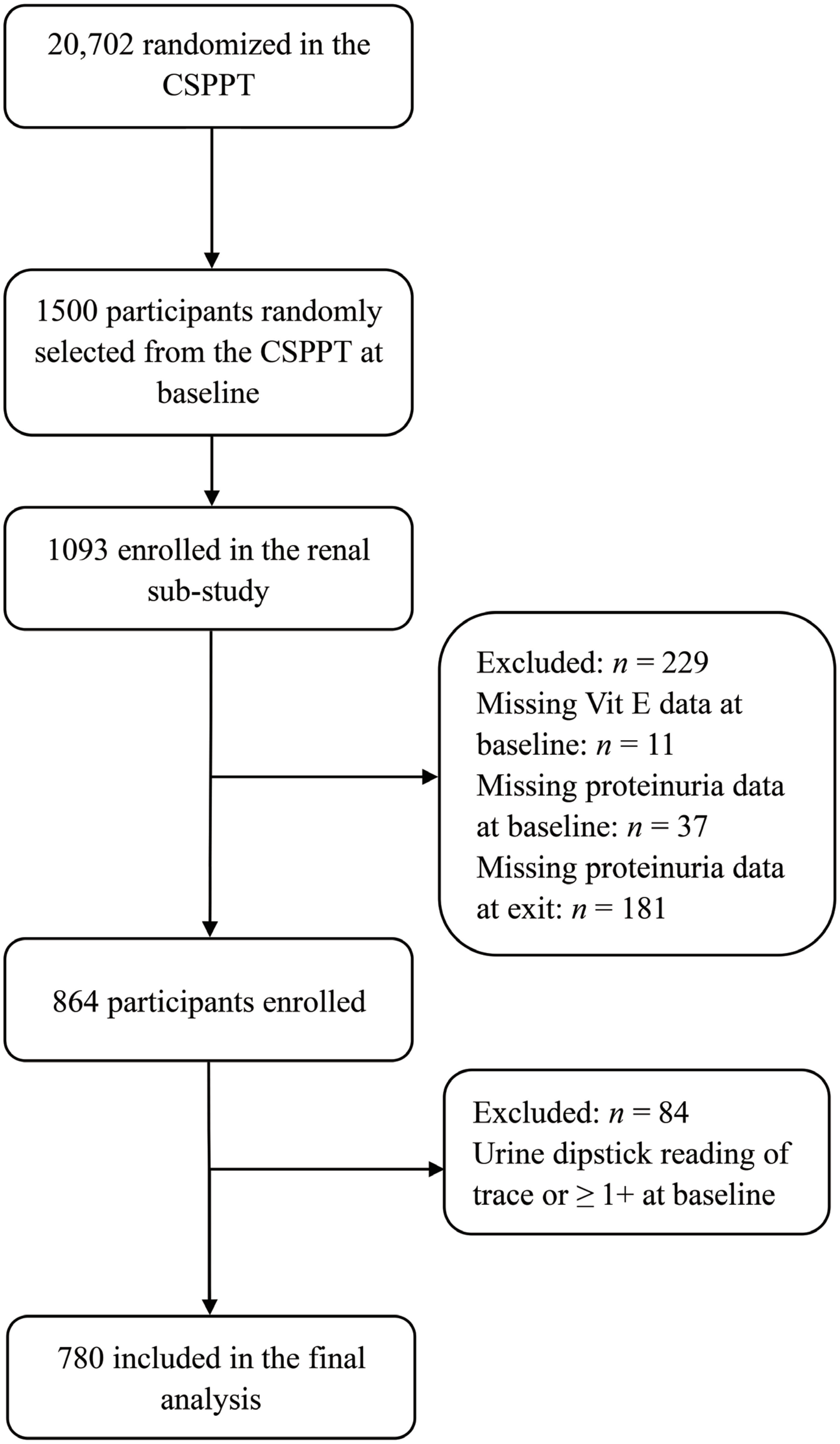


**Supplemental Figure 1: Flow chart of the participants. CSPPT: China Stroke Primary Prevention Trial.**

**Supplemental Table 1: Characteristics of the included and excluded populations in the current study**

| Characteristics | Total (*n* = 1093) | Included (*n* = 780) | Excluded (*n* = 313) |
|---|---|---|---|
| Male, *n* (%) | 435 (39.8) | 281 (36.0) | 154 (49.2) |
| Age, years | 59.5 (7.6) | 59.5 (7.6) | 59.6 (7.8) |
| BMI, kg/m$^2$ | 25.6 (3.6) | 25.6 (3.6) | 25.6 (3.7) |
| BP, mmHg | | | |
| Baseline SBP | 166.5 (19.9) | 165.7 (19.3) | 168.6 (21.2) |
| Baseline DBP | 94.4 (11.8) | 93.9 (11.8) | 95.6 (11.8) |
| Time-averaged SBP | 139.8 (10.9) | 139.0 (10.1) | 141.9 (12.5) |
| Time-averaged DBP | 83.5 (7.4) | 83.1 (7.1) | 84.4 (7.9) |
| Enalapril–folic acid, *n* (%) | 537 (49.1) | 389 (49.9) | 148 (47.3) |
| Current smoking, *n* (%) | 249 (22.8) | 170 (21.8) | 79 (25.2) |
| Current alcohol drinking, *n* (%) | 254 (23.3) | 168 (21.6) | 86 (27.5) |
| Laboratory results | | | |
| Triglycerides, mmol/L | 1.7 (0.9) | 1.7 (0.9) | 1.7 (0.9) |
| Total cholesterol, mmol/L | 5.7 (1.1) | 5.6 (1.1) | 5.7 (1.2) |
| HDL-C, mmol/L | 1.3 (0.4) | 1.3 (0.4) | 1.3 (0.4) |
| Glucose, mmol/L | 6.0 (1.8) | 5.9 (1.7) | 6.1 (1.9) |
| Homocysteine, µmol/L | 14.7 (9.1) | 14.4 (8.6) | 15.5 (10.0) |
| Folate, ng/mL | 7.6 (3.0) | 7.6 (3.0) | 7.5 (3.0) |
| Vitamin B12, pg/mL | 404.2 (146.0) | 401.9 (150.0) | 409.8 (135.4) |
| eGFR, mL/min/1.73m$^2$ | 93.9 (12.7) | 94.7 (11.9) | 92.0 (14.5) |
| Medication use, *n* (%) | | | |
| Antihypertensive drugs | 537 (49.1) | 377 (48.3) | 160 (51.1) |
| Lipid-lowering drugs | 6 (0.5) | 2 (0.3) | 4 (1.3) |
| Glucose-lowering drugs | 17 (1.6) | 11 (1.4) | 6 (1.9) |
| Antiplatelet drugs | 49 (4.5) | 34 (4.4) | 15 (4.8) |

Variables are presented as mean (SD) or *n* (%). BP: blood pressure; SBP: systolic blood pressure; DBP: diastolic blood pressure; eGFR: estimated glomerular filtration rate; HDL-C: high-density lipoprotein cholesterol; SD: standard deviation; BMI: body mass index.

**Supplemental Table 2: Baseline characteristics of the study participants by treatment groups**

| Characteristics | Enalapril ($n$ = 391) | Enalapril-folic acid ($n$ = 389) | $P$-value |
|---|---|---|---|
| Male, $n$ (%) | 141 (36.1) | 140 (36.0) | 0.983 |
| Age, years | 59.6 (7.5) | 59.4 (7.6) | 0.717 |
| BMI, kg/m$^2$ | 25.3 (3.6) | 25.8 (3.6) | 0.073 |
| BP, mmHg | | | |
| Baseline SBP | 165.4 (18.7) | 166.0 (19.8) | 0.644 |
| Baseline DBP | 93.4 (12.0) | 94.5 (11.6) | 0.214 |
| Time-averaged SBP | 138.9 (9.8) | 139.0 (10.4) | 0.925 |
| Time-averaged DBP | 82.9 (7.1) | 83.3 (7.1) | 0.350 |
| Current smoking, $n$ (%) | 82 (21.0) | 88 (22.6) | 0.687 |
| Current alcohol drinking, $n$ (%) | 91 (23.3) | 77 (19.8) | 0.398 |
| Laboratory results | | | |
| Triglycerides, mmol/L | 1.7 (0.9) | 1.7 (0.9) | 0.880 |
| Total cholesterol, mmol/L | 5.6 (1.1) | 5.7 (1.1) | 0.225 |
| HDL-C, mmol/L | 1.3 (0.4) | 1.3 (0.4) | 0.681 |
| Glucose, mmol/L | 5.9 (1.6) | 6.0 (1.9) | 0.494 |
| Total homocysteine, μmol/L | 14.3 (8.1) | 14.5 (9.2) | 0.805 |
| Folate, ng/mL | 7.6 (3.0) | 7.7 (3.1) | 0.643 |
| Vitamin B12, pg/mL | 392.8 (132.7) | 411.1 (165.3) | 0.090 |
| eGFR, mL/min/1.73m$^2$ | 95.0 (10.9) | 94.4 (12.8) | 0.465 |
| Medication use, $n$ (%) | | | |
| Antihypertensive drugs | 198 (50.6) | 179 (46.0) | 0.196 |
| Lipid-lowering drugs | 1 (0.3) | 1 (0.3) | 1.000 |
| Glucose-lowering drugs | 5 (1.3) | 6 (1.5) | 0.755 |
| Antiplatelet drugs | 17 (4.3) | 17 (4.4) | 0.988 |

Variables are presented as mean (SD) or $n$ (%). BP: blood pressure; SBP: systolic blood pressure; DBP: diastolic blood pressure; eGFR: estimated glomerular filtration rate; HDL-C: high-density lipoprotein cholesterol; SD: standard deviation; BMI: body mass index.

**Supplemental Table 3: Baseline characteristics of the study participants by exit proteinuria levels**

| Characteristics | None (*n* =688) | Trace (*n* = 63) | ≥1+ (*n* = 29) |
|---|---|---|---|
| Age, years | 59.5 (7.4) | 59.1 (8.0) | 60.3 (9.4) |
| Male, *n* (%) | 245 (35.6) | 24 (38.1) | 12 (41.4) |
| BMI, kg/m$^2$ | 25.6 (3.6) | 25.5 (3.6) | 25.4 (3.8) |
| BP, mmHg | | | |
| Baseline SBP | 165.5 (19.5) | 165.6 (16.7) | 170.0 (18.2) |
| Baseline DBP | 94.0 (11.8) | 93.7 (12.6) | 92.9 (11.2) |
| Time-averaged SBP | 139.0 (9.8) | 138.2 (11.7) | 139.3 (12.3) |
| Time-averaged DBP | 83.2 (7.1) | 82.7 (6.8) | 82.1 (7.7) |
| Enalapril–folic acid, *n* (%) | 339 (49.3) | 36 (57.1) | 14 (48.3) |
| Current smoking, *n* (%) | 150 (21.8) | 15 (23.8) | 5 (17.2) |
| Current alcohol drinking, *n* (%) | 147 (21.4) | 16 (25.4) | 5 (17.2) |
| Laboratory results | | | |
| Vitamin E, μg/mL | 9.6 (3.5) | 8.7 (2.8) | 8.9 (2.6) |
| Triglycerides, mmol/L | 1.7 (0.9) | 1.5 (0.7) | 1.9 (1.0) |
| Total cholesterol, mmol/L | 5.7 (1.1) | 5.5 (1.0) | 5.8 (1.2) |
| HDL-C, mmol/L | 1.3 (0.4) | 1.4 (0.3) | 1.4 (0.4) |
| Glucose, mmol/L | 5.9 (1.6) | 6.1 (2.1) | 6.2 (3.0) |
| Total homocysteine, μmol/L | 14.5 (8.8) | 13.3 (7.2) | 13.8 (6.4) |
| Folate, ng/mL | 7.7 (3.1) | 7.6 (3.2) | 6.8 (2.2) |
| Vitamin B12, pg/mL | 403.2 (137.5) | 400.0 (260.4) | 376.4 (108.9) |
| eGFR, mL/min/1.73m$^2$ | 94.6 (11.9) | 95.2 (10.8) | 95.0 (13.5) |
| Medication use, *n* (%) | | | |
| Antihypertensive drugs | 335 (48.7) | 30 (47.6) | 12 (41.4) |
| Lipid-lowering drugs | 2 (0.3) | 0 | 0 |
| Glucose-lowering drugs | 8 (1.2) | 2 (3.2) | 1 (3.4) |
| Antiplatelet drugs | 30 (4.4) | 2 (3.2) | 2 (6.9) |

Variables are presented as mean (SD) or *n* (%). SBP: systolic blood pressure; DBP: diastolic blood pressure; eGFR: estimated glomerular filtration rate; HDL-C: high-density lipoprotein cholesterol; SD: standard deviation; BMI: body mass index.

**Supplemental Table 4: Concomitant medications during the treatment period by vitamin E quartiles* (*n* [%])**

| Medication | Total | Q1 (< 7.3 μg/mL) | Q2 (7.3–<8.8 μg/mL) | Q3 (8.8–<11.3 μg/mL) | Q4 (≥ 11.3 μg/mL) | *P*-value |
|---|---|---|---|---|---|---|
| Calcium channel blockers | 624 (80.0) | 161 (82.6) | 152 (77.9) | 158 (81.0) | 153 (78.5) | 0.630 |
| Diuretics | 476 (61.0) | 131 (67.2) | 121 (62.1) | 120 (61.5) | 104 (53.3) | 0.045 |
| Lipid-lowering drugs | 1 (0.1) | 0 (0.0) | 1 (0.5) | 0 (0.0) | 0 (0.0) | 1.000 |
| Glucose-lowering drugs | 14 (1.8) | 2 (1.0) | 6 (3.1) | 2 (1.0) | 4 (2.1) | 0.410 |
| Antiplatelet drugs | 5 (0.6) | 1 (0.5) | 1 (0.5) | 2 (1.0) | 1 (0.5) | 1.000 |

*Regular concomitant medication was defined as ≥ 180 cumulative days of taking the drug of interest.

**Supplemental Table 5: The association between plasma vitamin E and the development of proteinuria with further adjustment for the use of diuretics during the treatment period**

| Vitamin E, μg/mL | *n* | Events (%) | Crude models | | Adjusted models* | |
|---|---|---|---|---|---|---|
| | | | OR (95% CI) | *P*-value | OR (95% CI) | *P*-value |
| Continuous (per SD increment) | 780 | 92 (11.8) | 0.77 (0.60, 0.98) | 0.033 | 0.73 (0.56, 0.97) | 0.027 |
| Quartiles | | | | | | |
| Q1 (< 7.3) | 195 | 31 (15.9) | reference | — | reference | — |
| Q2 (7.3–<8.8) | 195 | 21 (10.8) | 0.64 (0.35, 1.16) | 0.138 | 0.59 (0.32, 1.09) | 0.094 |
| Q3 (8.8–<11.3) | 195 | 20 (10.3) | 0.60 (0.33, 1.10) | 0.101 | 0.58 (0.31, 1.11) | 0.100 |
| Q4 (≥ 11.3) | 195 | 20 (10.3) | 0.60 (0.33, 1.10) | 0.101 | 0.56 (0.29, 1.11) | 0.097 |
| Categories | | | | | | |
| Q1 (< 7.3) | 195 | 31 (15.9) | reference | — | reference | — |
| Q2–Q4 (≥ 7.3) | 585 | 61 (10.4) | 0.62 (0.39, 0.98) | 0.042 | 0.58 (0.35, 1.00) | 0.038 |

*Adjusted for age, sex, body mass index, treatment group, smoking status, alcohol intake, SBP, eGFR, fasting glucose, total cholesterol, triglycerides, high-density lipoprotein cholesterol, total homocysteine, use of antihypertensive drugs at baseline, and time-averaged SBP during treatment. SBP: systolic blood pressure; eGFR: estimated glomerular filtration rate; OR: odds ratio; CI: confidence interval; SD: standard deviation.
